# Developing the ‘gripes’ tool for junior doctors to report concerns: a pilot study

**DOI:** 10.1186/s40814-016-0100-0

**Published:** 2016-09-29

**Authors:** S. Carr, T Mukherjee, A. Montgomery, M. Durbridge, C. Tarrant

**Affiliations:** 1 Department of Clinical Education, University Hospitals of Leicester, Leicester, UK; 2 University Hospitals of Leicester, Leicester, UK; 3 Queen’s University Belfast, Belfast, UK; 4 Department of Health Sciences, University of Leicester, Leicester, UK

## Abstract

**Background:**

Junior doctors often have concerns about quality and safety but show low levels of engagement with incident reporting systems. We aimed to develop and pilot a web-based reporting tool for junior doctors to proactively report concerns about quality and safety of care, and optimise it for future use.

**Methods:**

We developed the gripes tool with input from junior doctors and piloted it at a large UK teaching hospital trust. We evaluated the tool through an analysis of concerns reported over a 3-month pilot period, and through interviews with five stakeholders and two focus groups with medical students and junior doctors about their views of the tool.

**Results:**

Junior doctors reported 111 concerns during piloting, including a number of problems previously unknown to the trust. Junior doctors felt the tool was easy to use and encouraged them to report. Barriers to engagement included lack of motivation of junior doctors to report concerns, and fear of repercussions. Ensuring transparency about who would see reported concerns, and providing feedback across whole cohorts of junior doctors about concerns raised and how these had been addressed to improve patient safety at the trust, were seen having the potential to mitigate against these barriers. Sustainability of the tool was seen as requiring a revised model of staffing to share the load for responding to concerns and ongoing efforts to integrate the tool and data with other local systems for gathering intelligence about risks and incidents. Following piloting the trust committed to continuing to operate the gripes tool on an ongoing basis.

**Conclusions:**

The gripes tool has the potential to enable trusts to proactively monitor and address risks to patient safety, but sustainability is likely to be dependent on organisational commitment to staffing the system and perceptions of added value over the longer term.

**Electronic supplementary material:**

The online version of this article (doi:10.1186/s40814-016-0100-0) contains supplementary material, which is available to authorized users.

## Background

For healthcare organisations to learn, and to proactively improve quality and safety of patient care, it is essential to enable healthcare staff to report incidents and concerns [1]. In the wake of the Francis enquiry in the UK, particular attention has been focused on efforts to support staff in raising concerns about patient safety [2, 3], with junior doctors being identified as having an important role to play as the ‘eyes and ears’ of the NHS [4]. Junior doctors are in a unique position to identify concerns about patient safety: they spend much of their time in clinics or on hospital wards and are closely involved in day to day care of patients. They may also become aware of wider organisational problems that impact across different wards or units within hospitals and may be more likely to notice unacceptable practices [4]. Although junior doctors often have concerns about quality and safety, they show low levels of engagement with incident reporting systems [5, 6].

Developing a system which encourages junior doctors to report their concerns to the organisations in which they are training requires an awareness of barriers to reporting and features of a system which might make doctors more likely to engage [7]. The most common disincentives to reporting concerns described by junior doctors are a lack of belief in local reporting systems, a perception that local reporting does not result in any action, a lack of role modelling by senior doctors, uncertainty about what to report, inflexibility of reporting systems, and lack of feedback [8, 9]. The high-profile negative treatment of whistleblowers in the NHS is a potential barrier to raising concerns [3]. More broadly, fear of blame or repercussions, acceptance of errors as inevitable, and rejection of managerial scrutiny are also recognised as significant barriers to reporting [10, 11].

The GMC’s national training survey in the UK is a route through which junior doctors can raise concerns about patient safety in the organisations in which they are training [12]. In 2013, 5.2 % of responders raised concerns and 28 % of these concerns were new or unknown to the organisations involved. In 2014, partly due to changes to the wording of the survey, only 0.8 % of junior doctors reported concerns but over half of these were previously unknown to the organisations involved [8]. With recognition that there would be value in encouraging junior doctors to report their concerns directly with the organisation in which they were training, rather than reporting retrospectively through the training survey, we aimed to develop and evaluate a web-based reporting tool for junior doctors to proactively report concerns about quality and safety.

Incident reporting systems are used extensively across healthcare settings [13]; for example, all trusts in England have systems through which staff are required to report errors and adverse events, and trusts are required to upload reported incidents to the National Reporting and Learning System (NRLS) [14]. These systems are, however, targeted at capturing ‘serious’ incidents, errors, and near misses and are not designed to capture the full range of concerns that staff may have about quality and safety of care. Improving patient safety requires going beyond a reactive approach to mistakes. A more proactive monitoring of concerns is likely to generate a deeper understanding of the local organisational influences and preconditions which may present risks to patient safety, and improves organisational capacity for learning, by presenting opportunities to intervene in problems before they escalate and result in actual harm to patients [15, 16].

We conducted a pilot study to field test the tool, to identify barriers and facilitators to successful implementation, and to gain insight into how to optimise for future use the tool and systems for responding to concerns.

## Methods

### Baseline

The participating trust was a large teaching hospital trust located in a city in the Midlands of England. The trust had three hospital sites in different locations, with between 700 and 800 junior doctors working in the trust. The trust had a number of reporting systems already in place including the Datix incident reporting system, a staff concerns reporting telephone line, an online forum ‘The Staff Room’, directors’ breakfasts, a bullying and harassment line, and a whistleblowing policy.

### Intervention development

We developed the gripes tool for junior doctors to report concerns (a ‘gripe’ is defined in the Oxford English Dictionary as ‘a minor complaint’). This comprised an online reporting form and an associated system for responding to concerns. The form was available via the trust website which doctors could access whenever they logged into a trust computer and included fields to select category of concern (lack of staffing resources; IT problems; problems of quality of care; problems with patient management and flow; training or supervision; communication or information transfer; teamwork or working culture; problems with care processes, policies, or guidelines; equipment problems; ward environment; other) as well as a text field to describe the concern. The design of the form and the choice of categories on concern to include in the form were informed by preliminary focus groups with junior doctors about their views on reporting concerns [9]. The tool was promoted to junior doctors through design of a gripes logo, posters placed in clinical areas, pop-up advertising on the main trust website, and face-to-face promotion in junior doctor meetings and walk-rounds across the trust. The submission system for the form was set up to allow anonymised reporting with no personal details or information about usernames being attached to the report, but junior doctors could choose to include their email address in the report in order to get a response if they wished.

A three-person team managed the gripes system and took daily responsibility for monitoring and responding to the concerns reported. The team comprised a registrar seconded on a clinical education fellowship with dedicated time away from clinical duties; the director of safety and risk at the trust (an executive role that involves taking responsibility and providing clinical leadership for safety across the trust); and an information analyst. Prompt feedback about actions taken in response to concerns was provided to individual junior doctors who reported concerns, if contact details were provided. Concerns were resolved by the gripes team where possible or escalated by team through various routes (e.g. through IT services, the trust executive quality board, the trust chief executive).

The tool was piloted at the participating trust from 8 February to 8 May 2015. The Template for Intervention Description and Replication (TIDieR) checklist [17] (Additional file 1) provides a full description of the intervention and its implementation.

### Evaluation

We evaluated the gripes tool through analysis of reported concerns and a qualitative evaluation to optimise the intervention and to explore barriers and facilitators to implementation.

Concerns were analysed descriptively, to summarise the number reported, and patterns of reporting. We interviewed five stakeholders (members of the gripes team and trust senior executives) about their experiences of operating the system and managing concerns. We conducted two focus groups with nine participants overall (one specialty trainee, one core trainee, two foundation year 2 doctors, one foundation year 1 doctor, and four final year medical students) to examine user views of the tool and how it could be improved. Participants were recruited by email using local lists and snowball sampling. Informed consent was gained from participants. Focus groups were facilitated by TM and CT, with a topic guide used flexibly to promote discussion. Interviews and focus groups were digitally recorded and transcribed verbatim.

Qualitative data from the focus groups and interviews were analysed thematically [18]. Initial reading and open-coding of a selection of transcripts was used to develop a coding frame; this was applied to the full data set using NVivo 10. The coding frame was revised iteratively, with revisions informed by discussions within the research team. In presenting findings, we attribute quotations to either stakeholders (SH) or focus group participants (FG).

## Results

### Concerns reported

**Fig. 1:**
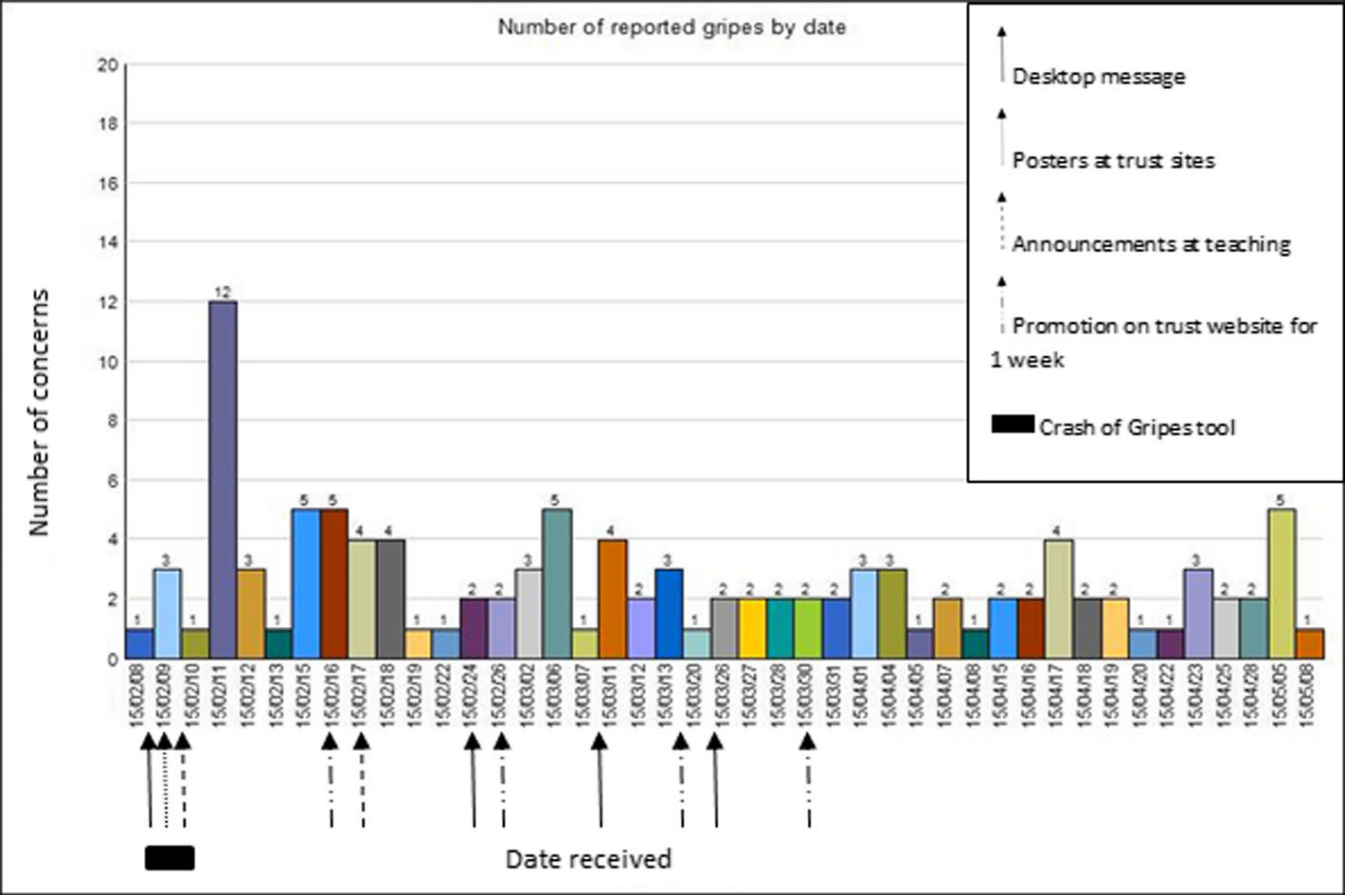
Reported concerns by date

The gripes website attracted over 1500 page views during the pilot period. Overall, 111 concerns were reported. Figure 1 shows the number of concerns reported by date across this time period alongside a variety of activities promoting the tool.

Most concerns were flagged as coming under one or two categories on the form; six concerns were reported as falling into four or more categories (Table 1). All concerns categorised as ‘other’ were also categorised in one of the other categories.

**Table 1:** Categories of concerns

Category of concern	No. of times category selected
1. Lack of staffing resources	36
2. IT problems	30
3. Problems of quality of care	17
4. Problems with patient management and flow	15
5. Training or supervision	15
6. Communication or information transfer	13
7. Teamwork or working culture	12
8. Problems with care processes, policies, or guidelines	12
9. Equipment problems	12
10. Ward environment	7
11. Other (e.g. problem with access cards, suggestion for revision to clerking proformas)	9

The gripes team categorised four concerns that had been reported through the gripes tool as ‘incidents’ that should have been reported via the trust’s official incident reporting system. These included reports of falls and a medication error. The team identified that all four concerns had also been reported as incidents. As such, there was no evidence that the introduction of the gripes tool had resulted in incidents going unreported through the appropriate system.

In responding to these reported concerns, the gripes team sorted the concerns into groups, so that they could be passed onto relevant individuals or departments within the trust to be dealt with. The groups, and the corresponding proportion of concerns, were the following: ‘information, management, and technology’ (32 %, *n* = 36); ‘staffing and rotas’ (28 %, *n* = 31); ‘patient care concerns’ (23 %, *n* = 26); ‘organisational issues’ (9 %, *n* = 10); and ‘equipment problems’ (4 %, *n* = 4).

The gripes team was able to quickly rectify a number of urgent or straightforward problems, such as missing or broken equipment, and some personal issues such as difficulties with annual leave. More complex or serious problems were escalated within the trust. Examples of serious problems that the team was alerted to, and was able to address, included the following:

1. Following a bank holiday weekend, several doctors reported inadequate patient reviews for outlying patients on certain wards. The concern was escalated to senior management and quickly rectified without detriment to patient safety. A plan was created to avoid future reoccurrences.
2. Several locum doctors reported that they had no access to electronic prescribing systems and investigations, which affected their ability to work safely and efficiently. The junior doctor administrators were alerted to this and amended the induction process for temporary staff joining the trust.

### Optimising the tool: engaging junior doctors

Focus group participants highlighted a number of ways to optimise the gripes tool and system to help promote junior doctor engagement, including further simplification of the form, promotion of the tool and feedback of actions taken in response to concerns, transparency about who would see reported concerns, and consideration of making the tool available off trust premises.

### Further simplification of the gripes form

Junior doctors felt that the form made it easy for them to report a broad range of concerns, which could not be classified or easily reported within the constraints of existing incident reporting systems. They argued that the number of categories could be reduced and that this would not only make the form clearer and easier to use but also would generate better quality information.

I think the less categories maybe the better the quality of gripe you’ll get. (FG)

### Promotion of the tool

Promoting the tool to junior doctors across the multi-site trust proved difficult; the highest levels of reporting came from the site at which the gripes team registrar was located and had been able to do the most face-to-face promotional work (83 reports, as compared to a total of 28 across the other two sites). Focus group participants indicated that they responded best to personal contact and suggested putting posters up in doctors’ offices, promoting the GRIPE tool in induction and training sessions, and getting senior staff to encourage their juniors to use the tool.

If you were to have a consultant […] to mention to the doctors on the ward that if there are unsatisfactory things we now have this new tool. (FG)

The idea of having ‘gripes champions’ was also raised as a way of promoting engagement and making sure concerns were heard, acted upon, and feedback.

You could have for each group of doctors, like for the F1s [doctors in foundation year training], they have an F1 gripe champion. They get sent all the F1 gripes, and then they have a role where they regularly liaise with [senior staff] and say ‘these are the gripes we’re receiving’ (FG)

### Listening and responding to concerns

The gripes team registrar reviewed concerns daily, provided prompt feedback to all who provided their contact details, and where possible worked with the junior doctor who reported the concern to try to resolve the problem. The trust director of safety and risk also reviewed concerns regularly and, in consultation with the registrar, took responsibility for escalating more serious or complex problems: working with other senior staff in the trust to coordinate solutions and develop action plans and reporting back to higher levels of the organisation.

Junior doctors were extremely positive about the way the team responded: they felt they were being listened to and taken seriously and saw this as a key for their engagement with the gripes tool.

For my friend that was the power of it, she’s told me about it, because […] she got a real response from a real person. She was like ‘oh, they responded’. (FG)

Receiving feedback that concerns were being listened to and acted upon was seen to be extremely important in motivating engagement with the tool. Finding ways of going beyond feedback to individuals, to provide feedback to whole cohorts of junior doctors across the trust about how reporting was making a difference at the trust, was seen as key to getting broad engagement.

If you were sort of saying there is a GRIPE tool, this is how you can get hold of it, look what we’ve done in the past however months, then that would kind of encourage. [You could do it] at that induction teaching session (FG)

If people see results and things are resolved because of it, then they will use it. (FG)

### Anonymity and confidentiality

Focus group participants liked the option of having the choice of providing contact details with their reported concern or reporting anonymously. Although raising concerns was seen as less threatening than reporting incidents, participants still suggested that fear of who might see the concerns and the risk that reporting could result in negative repercussions were barriers to engagement. It was seen as difficult to ensure full confidentiality if, for example, there was only one junior doctor working on a ward about which a concern was raised. Participants reported that they would like to know who was operating the system and who would see their reports and felt they would be more likely to engage with a system that was being operated by experienced trainee doctors.

That’s probably one of the biggest barriers. […] A reason I wouldn’t use it, is the fear of where it might go, […] maybe I wouldn’t use it because I’d be a bit afraid of what might happen because of what I said. (FG)

I’d like to know that there are a group of junior doctors […] people like me, who can relate to my problems. […] ‘We’re junior doctors as well, we’ll listen to your gripes and we’ll do something about it.’ (FG)

Concerns about negative consequences of reporting were expected to be relieved to some extent if the gripes tool became embedded in the organisation, as long as there was good evidence that concerns were being handled sensitively by the gripes team, and evidence that reporting actually did make a difference.

The more it annoys you and you actually, to an extent actually think you might be fearful of saying it, that makes it even more important you actually do report it. So I think for me it’s actually knowing something is coming out of this, otherwise I’m not wasting my time. (FG)

### Accessibility of the tool

Although the tool was made available to all junior doctors via any trust computer, focus group participants felt there would be value in looking at ways of enabling people to report concerns outside of the ward environment and from home. The idea of an app was popular, but participants also suggested using email, text messages, and an external website.

Could you gripe once you have got back home after a shift? Because obviously you can't access that from home. […] Probably email, […] or an app accessible from home. (FG)

### Optimising the tool: considerations of value and sustainability

Stakeholders particularly reflected on the value of continued use of the gripes tool and on optimising the sustainability of models for operating the system.

### Value of the tool: part of a bigger picture

The knowledge generated through the gripes tool was seen by stakeholders as adding value to the knowledge they already had access to through existing trust reporting systems. Pre-emptive reporting of problems by junior doctors was seen as adding a new perspective, highlighting problems at the front line of care that were unknown to the trust, and enabling problems to be tackled in a timely way before they escalated. One stakeholder described the potential of the tool to act as a ‘barometer’, pointing to areas where concerns were clustered.

I think it’s been useful because I know we’ve identified some safety concerns that we didn’t know about before. […] There’s things come up about staffing in areas that we didn’t know were a problem. (SH)

[It can be] a sort of barometer […] to say Ward X is getting hot, something is happening on Ward X. […], or we’re getting a lot of things about rotas. (SH)

One concern expressed by stakeholders was whether, over time, the same issues would emerge repeatedly meaning that they were investing time and resources into a system that did not generate new information. On the other hand, stakeholders recognised that running the tool over time could help them see whether problems had been resolved and which issues persisted.

What’s a long term value going forward if we’re just seeing the same things reported? I guess if we’re not seeing, if we are seeing the same things reported we haven’t remedied them. (SH)

Integrating the data from gripes, and systems for responding to reported concerns, with other trust data and reporting systems, was seen to be key to maximising the value of the system.

We also do one paper every month which is triangulating all of those eight [reporting mechanisms] together, and it says these are the things that have been reported through all of those sources, and these are the top things that seem to be coming out. (SH)

### Sustainability: staffing the system

Designing a sustainable system was challenging. The day to day running of the system was dependent on a registrar in a clinical education fellow post; the gripes register left the trust after the pilot was completed, but the trusts were able to reallocate this role to a new incoming fellow to ensure the work continued. IT and analytic support were also important but hard to resource. The level of commitment of the trust director of safety and risk was seen as unsustainable beyond the pilot, partly because it was not financially resourced. The proposed solution was to gain the commitment of other trust directors to share the responsibility for managing reported concerns, rather than this responsibility resting solely with a single director.

I think it would be incredibly difficult to sustain it within current resources, because essentially most of it, [we] have been doing it, we’ve been doing it in our own time. (SH)

[We’ve] discussed a model whereby when the concerns come in they get allocated to an associate medical director with that portfolio […]. So the issues will be sent to the relevant associate medical director, with [director of safety and risk]’s team keeping an oversight of all the issues. (SH)

Stakeholders also discussed the possibility of running the gripes tool intermittently—for example, having a ‘gripes week’ during each rotation of junior doctors. Focus group participants argued, however, that there were significant benefits to keeping the gripes reporting tool live even if the team were not always able to provide an immediate response.

I think it has to be permanently open. […] I think the live thing in the shorter term is definitely more preferable because it improves your trust in that something is happening. If you actually get it embedded in the culture there would be no problem with [a delay in feedback] because you will be having positive feedback from things that have happened before. (FG)

### The problem of concerns that are not easily resolved

Stakeholders highlighted the problem of how to handle reported concerns that were valid but were difficult or impossible for the trust to resolve—either because they related to a longer term project or they were outside the control of the trust. They recognised a risk of being perceived to be ignoring these concerns or not taking them seriously and felt this could impact negatively on engagement with the system. How feedback was handled was seen as critical to this and stakeholders recognised that they may need to consider strategically how they managed this.

The response to the IT concerns is going to be disappointing to the doctors. Because until we put in the new electronic patient record, and the […] investment that comes with that […] it is unlikely that the users are going to see a big change in their experience. So what you are going to end up saying, sending them back is that we are really sorry, we know the IT systems aren’t very good, there is not a lot we can do about it at the moment, and that, those kind of responses are a tricky one. (SH)

## Discussion

This study involved the development and evaluation of a tool for junior doctors to report concerns about quality or safety of care to the organisation in which they were training. The tool generated 111 reports of concerns during the 3-month pilot period. Junior doctors may have a unique perspective on quality and safety problems, but there are also particular challenges in engaging them in reporting concerns to their training organisation. Our evaluation demonstrated that a well-designed reporting system can overcome barriers for junior doctors in reporting concerns about quality and safety of care. Using a simple form, and allowing junior doctors’ flexibility in what and how they reported, helped to address the oft-reported barrier that incident reporting systems are difficult and time-consuming to use [7] and enabled junior doctors to tell the organisation about the things that really mattered to them [9]. The trust has committed to running the system on an ongoing basis. The tool was relaunched following the pilot in December 2015, attracting a further 56 reported concerns between mid-December and mid-April 2016.

This pilot indicates that the gripes tool offers an approach for organisations to gather knowledge about risks to patient safety from junior doctors, who are well-placed to see these risks in the course of their day to day practice. Given that there are multiple tools and systems for reporting concerns in operation across hospitals, this is only one part of a bigger picture. Gripes data is likely to be of most value when triangulated or used alongside data on problems and incidents from a range of other sources including incident reporting systems and soft intelligence from staff and patients about problems in care [19].

Simply introducing another reporting form, however, without genuine commitment to working collaboratively with staff to ensure concerns are listened to and addressed [20], is unlikely to be effective and may even be counterproductive. As Macrae suggests, ‘the incident reports themselves do not matter nearly as much as the practical work of investigating and understanding a particular aspect of an organisational system and then working collaboratively to improve it’ [21]. The pilot highlighted the challenges inherent in efforts to implement a sustainable system for responding to concerns. During the pilot period, the trust director of safety and risk invested significant time in reviewing and dealing with more complex concerns. This level of commitment was seen as unsustainable beyond the pilot period, necessitating a change in approach for the longer-term roll out of the tool. The revised approach being taken by the trust going forward involves a review of each concern by the gripes registrar who deals with straightforward problems such as missing equipment. All concerns are forwarded to the trust director of safety and risk who monitors patterns of concerns, but also forwards more complex concerns to a team of six trust directors, each of whom takes responsibility for one of six categories of concern (lack of staffing resources; IT issues; training and supervision; equipment and ward environment; team work and communication; quality and safety of care). All IT related concerns are also sent directly to the trust IT department. Current plans in the UK to introduce ‘freedom to speak up’ guardians into NHS trusts offer another, as yet untested, option for responsibility for managing and responding to concerns [3].

Healthcare staff are less likely to engage with a reporting system if they do not receive feedback on the outcome of their reports or do not perceive that local reporting results in action [8, 9]. Correspondingly, participants in our study suggested that evidence that the system was making a difference—that concerns were being listened to and acted upon—was the most critical factor motivating them to use the tool. Ensuring the feedback reaching all junior doctors across the trust is likely to require multiple approaches used in combination [22]; the current gripes registrar is working on a project to develop viable approaches to feedback which will include email and face-to-face updates to the whole cohort of junior doctors at the trust, on concerns reported through gripes and actions taken.

This pilot study was limited in that it was run in a single large trust; the replicability of the gripes system in other contexts is likely to be in part dependent on the availability of resources to support its operation. Stakeholders suggested that the tool worked well because it was part of a wider shift within the trust towards developing a positive organisational climate of listening to staff concerns. The extent to which such a system will embed in other contexts is likely to be dependent on the local organisational climate in relation to listening to staff concerns.

## Conclusions

The use of a simple online reporting tool for junior doctors provides an approach for organisations to proactively detect problems in clinical systems at the frontline of care. The next steps will be assessing the sustainability of the system over an extended period of time, in particular, whether the system continues to generate new knowledge over time and continues to add value to other means of gathering intelligence about risk and incidents.

## Additional file

Additional file 1: TIDieR checklist for the gripes intervention. (DOCX 47 kb)
