# Supplementary material for: Developing the ‘gripes’ tool for junior doctors to report concerns: a pilot study
Source: Pilot Feasibility Stud. 2016 Sep 29;2:60. doi: 10.1186/s40814-016-0100-0 (PMC5108408; doi:10.1186/s40814-016-0100-0)
Supplement: Additional file 1: — TIDieR checklist for the gripes intervention. (DOCX 47 kb) [file 40814_2016_100_MOESM1_ESM.docx]

## Additional file 1: TIDieR checklist for the GRIPES intervention

|  | **Item** | Description |
| --- | --- | --- |
|  | **BRIEF NAME** | GRIPES: A tool for junior doctors to report concerns about quality and safety of care |
|  | **WHY** | Junior doctors often have concerns about quality and safety but show low levels of engagement with incident reporting systems. Common reasons why junior doctors do not speak up about concerns include difficult to use reporting systems, fear of retribution, and lack of visible change. Incident reporting systems do not facilitate proactive reporting of broader safety concerns. We aimed to develop a tool targeted at junior doctors that would encourage and enable them to report concerns. |
|  | **WHAT** | **Materials**:   - Online form for junior doctors (doctors in training) to use to report any concerns about quality or safety of care they encountered in daily practice. The form was short and simple. It included:   - Required fields – trust site; whether a trainee doctor; tick box category of concern (lack of staffing resources; IT problems; problems of quality of care; problems with patient management and flow; training or supervision; communication or information transfer; teamwork or working culture; problems with care processes, policies, or guidelines; equipment problems; ward environment; other); and text field to describe the concern.   - Optional fields – date; ward; and a field for junior doctors to include their email address if they would like a response (this field could be left blank for anonymity).   - A poster to promote the tool.   **Procedures**   - The tool was promoted to junior doctors through design of a GRIPES logo, posters placed in clinical areas, pop-up advertising on the main trust website, and face-to-face promotion in junior doctor meetings and walk-rounds across the trust. - The tool was ‘live’ and available for use by junior doctors to report concerns from 8 February – 8 May 2015. - The system was designed so that each concern reported was emailed to a GRIPES inbox which could be accessed by the GRIPES team. - Emails were reviewed by GRIPES team members, collated into a spreadsheet - Concerns were sorted into six categories to enable them to be passed on to relevant individuals or departments within the trust to be dealt with. The six categories were: information, management, and technology; staffing and rotas; patient care concerns; organisational issues; and equipment problems. - All individuals who included their email address on the form received a personalised reply from a member of the GRIPES team thanking them for their response and informing them of what would happen next. - The GRIPES team endeavoured to resolve any urgent, minor or straightforward problems immediately, and in consultation with the junior doctor concerned where appropriate. - The GRIPES team liaised with others (e.g. IT services, medical directors) to share and work towards resolving concerns. - More serious or complex concerns were escalated to higher levels of the organisation by the senior member of the GRIPES team. - Patterns of concerns were reviewed on an ongoing basis, and compared with issues raised through other trust reporting systems (e.g. DATIX). |
|  | **WHO PROVIDED** | Operating the system involved:   - Support from the trust IT service. This was critical in designing and maintaining the online reporting tool, and setting up promotional adverts on the trust website - A three-person team who managed the system and took daily responsibility for monitoring and responding to the concerns reported. The team comprised:   - A registrar seconded on a clinical education fellowship (and not working clinically in the trust) who promoted the tool to junior doctors, monitored concern emails daily, sent personalised reply emails to individuals who reported concerns, worked with junior doctors to resolve minor or straightforward concerns, liaised with the senior member of the GRIPES team about responding to concerns, and compiled feedback to post on the GRIPES website.   - An information analyst who was a member of the trust safety team and who imported concern emails into a spreadsheet, sorted concerns into categories and generated regular reports on number and type of concern, liaised with the senior member of the GRIPES team over responsibilities for responding to concerns, and liaised with IT with regard to IT-related concerns.   - The director of safety and risk at the trust who monitored concern emails daily, liaised with the registrar on the team over email responses to concerns, worked to support resolving minor or straightforward concerns, dealt with governance and maintained lines of communication and information about the project with senior levels of the trust, reviewed patterns of concerns in relation to data from other reporting systems within the trust, and liaised with other trust directors about plans for, and allocation of responsibilities for, responding to more serious and complex concerns. |
|  | **HOW** | - The GRIPES form could be accessed by junior doctors through the trust website, via any trust computer. Junior doctors could only access the tool while on trust premises. - The GRIPES team responded to individuals who gave their email address with personal feedback emails. - Generic feedback on the number and nature of concerns reported during the pilot period was provided on the GRIPES website. |
|  | **WHERE** | - The participating trust was a large teaching hospital trust located in a city in the Midlands of England. The trust had three hospital sites in different locations, and between 700 and 800 junior doctors working in the trust. - The trust had a number of reporting systems already in place including the DATIX incident reporting system, a staff concerns reporting telephone line, an online forum ‘The Staff Room’, directors’ breakfasts, a bullying and harassment line, and a whistleblowing policy. - Executive support and commitment to the project was high: the original idea for the project came from a consultation workshop with staff led by the trust chief executive. The trust’s director of safety and risk was a core and committed member of the GRIPES team. - The trust was perceived to have made big steps in recent years towards improving communication with, and listening to, staff, and establishing a ‘no blame’ culture. Senior staff were seen to be supportive of patient improvement work and open to criticism and to change. |
|  | **WHEN and**  **HOW MUCH** | - The GRIPES tool was live 24 hours a day, 7 days a week during the pilot period. - The GRIPES team monitored concerns and sent feedback emails on a daily basis, and worked to quickly follow up on concerns and get them resolved where possible; this sometimes required multiple phone calls, emails and meetings. - The estimated time spend on operating the system during the pilot period was:   - Registrar: varied depending on amount of promotional work and number of concerns being reported - up to two or three hours per day.   - Information analyst: one to two hours per week (but more time would be required to fully embed the system e.g. additionally recording allocation of responsibilities, actions and outcomes would increase his time needed to around half a day a week).   - Director: varied depending on number and severity of concerns – involved some work most days, adding up to around three to four hours a week. |
|  | **TAILORING** | Personal, tailored emails were sent to those who provided their email address. |
|  | **MODIFICATIONS** | Modifications made for the system re-launch in December 2015, including:   - Promotion of the tool during junior doctor induction, including feedback of concerns raised and actions taken - A change in personnel as the registrar is taking on a new role - Involvement of wider team of senior staff to manage responding to concerns   The possibility of making the tool available as an app is also under consideration. |
|  | **HOW WELL** | The GRIPES tool generated 111 concerns during the three month pilot period. The majority of these were seen by the GRIPES team as valid and appropriate concerns, and included a number of problems that were hitherto unknown to the trust. Four reports were deemed to count as ‘incidents’ rather than concerns, but these issues were identified as having also been reported on the trust incident reporting system. All but four reports came from trainee doctors.  Uptake of the tool was uneven across the three hospital sites, with 83 (75%) of concerns coming from one hospital site. This was the site at which the GRIPES team registrar was located, and had been able to do the most face-to-face promotional work, suggesting the need for more wide-reaching promotion of the tool.  Sustainability of the tool is dependent on commitment of resources to support its ongoing implementation. |
